# Editorial: Trust and Infrastructure in Scholarly Communications

**DOI:** 10.3389/frma.2022.925500

**Published:** 2022-06-01

**Authors:** Daniel W. Hook, Linda S. O'Brien, Stephen Pinfield

**Affiliations:** ^1^Digital Science, London, United Kingdom; ^2^Centre for Complexity Science, Imperial College London, London, United Kingdom; ^3^Department of Physics, Washington University in St Louis, St Louis, MO, United States; ^4^Griffith Business School, Griffith University, Southport, QLD, Australia; ^5^Information School, The University of Sheffield, Sheffield, United Kingdom

**Keywords:** trust, research infrastructure, scholarly communications, metrics, ranking, cultural norms

When Dickens wrote: “It was the best of times, it was the worst of times” in A Tale of Two Cities, he was describing the effects of two separate but linked revolutions—one in the UK where industrialization and technology had changed the social fabric as cities garnered population at the expense of their rural surroundings; and the other in France, were bloody revolution had overthrown the *ancien régime*, only to replace it with a new reign of terror. Today, we stand at the beginning of the first exponential industrial revolution: The technologies that have been born in the world of the Internet have elevated data and the AI that it fuels to the modern equivalents of oil and fire, respectively. We have seen this trend before in other industrial revolutions, but this time there is a difference—the technology that we have created has the capacity to design and build the technology that will supersede itself, leading to a self-fuelling feedback loop. Industrial revolutions have been inextricably linked to disruption—not just of industry, but also of society. The technical, economic, cultural, and societal structures that once seemed so embedded and immutable are changing quickly and the world of research is not immune.

An ever more connected system of global research information and infrastructure is transforming all aspects of the research process from how we discover and consume content to how we communicate it and the impact that it can have. At the same time, Web 2.0 has given the ability to self-publish to anyone—a tremendous freeing of global communication, but the signal-to-noise ratio has become challenging to manage, and fake news and unreliable information abound. In this new world research participation is becoming more global (both from international and intranational perspectives); research communication is more transparent through the open access, open data, and open science movements; it is becoming ever closer to translation and societal impact as we see through the rise in importance of the UN Sustainable Development Goals, a variety of grand challenge agendas, moonshots, and the adoption of impact into the evaluation environment. Data are at the centre of this change—whether it be the need to handle data volumes, the “shape” and format of data, or data as code and as a fuel for AI—the heart of any technology strategy is not just about how we collect, manipulate, consume and deploy data, but also about how it is structured, who can find it, who has access to it, who has sovereignty over it, and what capacity they have to calculate with it. And, it is more critical than ever that we understand the provenance, context, and bias of data. As data becomes part of our most powerful tools, we have to understand the “error bars” more than ever before. With so much change it is unsurprising that the infrastructures and norms of the research world, which were built in a different time, are under stress.

As a result of this impetus to change, infrastructures that have underpinned the research conversation for 350 years are changing and, as such, are vulnerable. There has never been a more important time to consider how we trust the infrastructures that we rely upon, and how those infrastructures can engender trust in communities. Thus, the collection of articles in this Research Topic reflect a diverse range of different perspectives on how the scholarly communications research infrastructure is changing and the issues of trust in both the best and worst of times.

Research metrics drive evaluations and reputations in many parts of the world. Gadd makes a powerful argument that institutions should challenge the current ranking methodology and introduce healthier approaches to ranking, while Sumner et al. consider a shift away from attention-based ranking and metrics by introducing measures of trust.

Carrying out research is also replete with challenges in a technology-driven age. The rise of essentially Western technologies carries with it implicit assumptions about how the world is structured. Zeitlyn considers issues of trust between researcher and research subject in the context of social anthropology when technology, used unwittingly, can compromise anonymity. Ruckstuhl considers related issues associated with indigenous populations and how Western ways of knowing are anathema to people who experience knowledge in a completely different way.

Flanagan et al. take a practitioners' view on how global norms simultaneously require and create the trust that is required to collaborate. Ignat et al. then question how that trust can be leveraged against us and undermined by surveillance capitalism in the research world. Porter imagines the stakeholders in the research community as citizens and conducts a large-scale analysis of the extent to which ORCID is an empowering and inclusive piece of infrastructure. Altman and Cohen take an expansive view and address the question of what makes a good ecosystem? Finally, Barbour discusses whether the disparate forces of openness, integrity, inclusion, and innovation in scholarly communication compete or complement each other.

We hope that this Research Topic leads to a broader and better-informed discussion on the infrastructures that are being created. It is clear that we are at a critical point in the development of the research world—setting up our infrastructures and cultural norms to respect many different perspectives will be critical in creating an inclusive and open yet trusted and sure foundation on which the future of research can be built.

## Author Contributions

All authors listed have made a substantial, direct, and intellectual contribution to the work and approved it for publication.

## Conflict of Interest

DH is the CEO of Digital Science, the provider of software in the research infrastructure space including Altmetric, Dimensions, Figshare, GRID, Overleaf, ReadCube, Ripeta, and Symplectic. The remaining authors declare that the research was conducted in the absence of any commercial or financial relationships that could be construed as a potential conflict of interest.

## Publisher's Note

All claims expressed in this article are solely those of the authors and do not necessarily represent those of their affiliated organizations, or those of the publisher, the editors and the reviewers. Any product that may be evaluated in this article, or claim that may be made by its manufacturer, is not guaranteed or endorsed by the publisher.

